# Revisiting Russell's Viper (*Daboia russelii*) Bite in Sri Lanka: Is Abdominal Pain an Early Feature of Systemic Envenoming?

**DOI:** 10.1371/journal.pone.0090198

**Published:** 2014-02-26

**Authors:** Senanayake A. M. Kularatne, Anjana Silva, Kosala Weerakoon, Kalana Maduwage, Chamara Walathara, Ranjith Paranagama, Suresh Mendis

**Affiliations:** 1 Department of Medicine, Faculty of Medicine, University of Peradeniya, Peradeniya, Sri Lanka; 2 Department of Parasitology, Faculty of Medicine and Allied Sciences, Rajarata University of Sri Lanka, Anuradhapura, Sri Lanka; 3 School of Medicine and Public Health, University of Newcastle, Callaghan, New South Wales, Australia; 4 Teaching Hospital, Anuradhapura, Sri Lanka; National University, Costa Rica

## Abstract

The Russell's viper (*Daboia russelii*) is responsible for 30–40% of all snakebites and the most number of life-threatening bites of any snake in Sri Lanka. The clinical profile of Russell's viper bite includes local swelling, coagulopathy, renal dysfunction and neuromuscular paralysis, based on which the syndromic diagnostic tools have been developed. The currently available Indian polyvalent antivenom is not very effective in treating Russell's viper bite patients in Sri Lanka and the decision regarding antivenom therapy is primarily driven by clinical and laboratory evidence of envenoming. The non-availability of early predictors of Russell's viper systemic envenoming is responsible for considerable delay in commencing antivenom. The objective of this study is to evaluate abdominal pain as an early feature of systemic envenoming following Russell's viper bites. We evaluated the clinical profile of Russell's viper bite patients admitted to a tertiary care centre in Sri Lanka. Fifty-five patients were proven Russell's viper bite victims who produced the biting snake, while one hundred and fifty-four were suspected to have been bitten by the same snake species. Coagulopathy (159, 76.1%), renal dysfunction (39, 18.7%), neuromuscular paralysis (146, 69.9%) and local envenoming (192, 91.9%) were seen in the victims, ranging from mono-systemic involvement to various combinations. Abdominal pain was present in 79.5% of these patients, appearing 5 minutes to 4 hours after the bite. The severity of the abdominal pain, assessed using a scoring system, correlated well with the severity of the coagulopathy (p<0.001) and the neurotoxicity (p<0.001). Its diagnostic validity to predict systemic envenoming is – Sensitivity 81.6%, Specificity 82.4%, Positive predictive value 91.2%. Thus, abdominal pain is an early clinical feature of systemic Russell's viper bite envenoming in Sri Lanka. However, it is best to judge abdominal pain together with other clinical manifestations on decision making.

## Introduction

The Russell's viper is considered a highly venomous snake throughout its range in Asia [1]. Based on the morphology and molecular evidence, the western and the eastern forms of this snake are now considered two separate species. The western form, *Daboia russelii* (Shaw and Nodder, 1797) is distributed throughout South Asia, west of the bay of Bengal, whereas the eastern form *Daboia siamensis* (Smith, 1917) is distributed in South-East Asia, east of the bay of Bengal [2]. Three sub species of the Russell's viper, *D. r. pulchella* (from Sri Lanka and southern India), *D. r. nordicus* (from northern India) and *D. r. russelii* (other areas) have been described from the regions to the west of the Bay of Bengal, based on morphology. Although these sub species have been placed under *Daboia russelii*, the taxonomic validity of this separation remains doubtful [2], [3].

**Figure 1:**
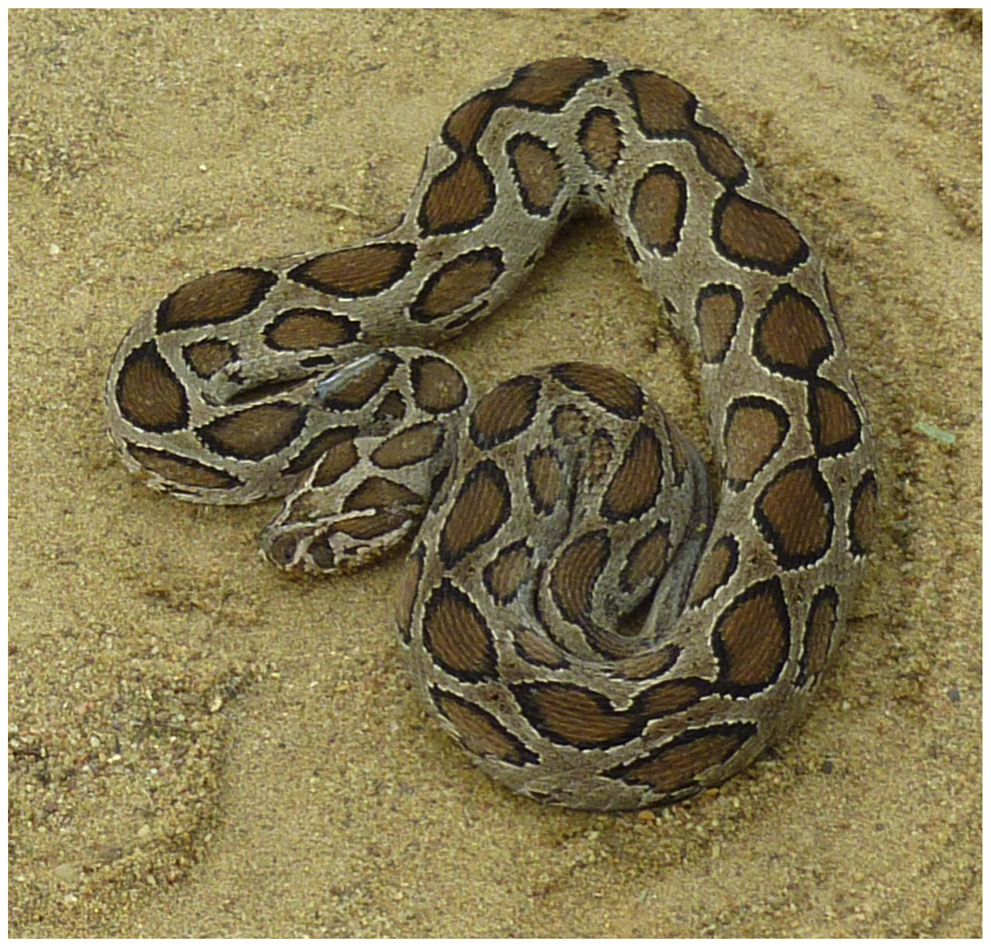
Russell's viper (*Daboia russelii*) adult specimen from Sri Lanka. Russell's vipers are distributed throughout Sri Lanka except at higher elevations (>1500 m) and are abundant in agricultural lands in the rural areas of the island's dry zone.

There are 37,000 snakebite admissions to Sri Lankan hospitals every year [4], [5]. The Russell's viper (Figure 1) is responsible for 30–40% of the snake bites, and to the most number of severe envenoming and fatalities compared to other snakes in Sri Lanka [5], [6]. Sri Lankans have been well aware of the aggressive nature and the lethality of the Russell's viper over many generations. In 1910, Abercromby [7] noted “…they [native Sri Lankans] consider the Russell's viper (Tic Polonga) as a personification of the devil.” Bites by Russell's vipers commonly occur in paddy (rice) fields and on footpaths at dusk and at dawn, affecting a large number of agricultural workers and so considered an occupational hazard in Sri Lanka [8].

The clinical profile of Russell's viper bites in Sri Lanka was first studied in detail by Jeyarajah in 1984 based on 22 cases [9], followed by a study of 23 cases by Phillips et al., in 1988 [10]. Kularatne [8] studied the epidemiology and clinical profile of Russell's viper bites in Sri Lanka based on 336 cases. Coagulopathy and acute kidney injury had been the major life threatening systemic manifestations of bites by this snake in Sri Lanka [8], [10]. Neuro-muscular paralysis, characterized by opthalmoplegia and ptosis, has been commonly observed in these with rare occurrence of respiratory muscle paralysis [8], [9]. Rhabdomyolysis [10], chronic renal failure [11], myocardial infarction [12] and secondary hypopituitarism [13] have also been reported in Russell's viper bite patients in Sri Lanka. Although generally not severe, local swelling has been a common feature seen in this country [8], [10].

Differences in venom composition between Sri Lankan and northern Indian Russell's vipers have been used to support separation into two subspecies [14]. An attempt at developing a monospecifc antivenom against the venom of the Sri Lankan Russell's viper was successful, but it did not go beyond clinical trials [15]. The only antivenom available for Russell's viper bites in Sri Lanka is Indian-made polyvalent antivenom which has been raised against the venoms of four Indian snakes including Russell's vipers. This Indian polyvalent antivenom has been shown to be poorly effective in treating Sri Lankan Russell's viper bites [10]. It is noteworthy that this clinico-epidemiological information which lays the foundation for further clinical research as well as affecting clinical management, relies heavily on a few studies only, the latest being published ten years ago [8], [10]. Based on the observation that the clinical profile of Russell's viper bites in Sri Lanka presented as unique clinical syndromes, epidemiological tools for the syndromic identification of Russell's viper bite envenoming have been developed [16], [17]. The availability of offending snake specimens in snakebite instances has been less than 30% [8], therefore this method has been useful in making a presumptive diagnosis of Russell's viper bite envenoming. The incidence of recognised clinical features of Russell's viper envenoming are local envenoming 92%, coagulopathy 77%, neurotoxicity 78% and renal failure 18%, either in various combinations or as isolated manifestations [8]. When the combinations of clinical features do not follow a described pattern, the application of the syndromic diagnosis method to identify the offending snake could lead to an erroneous conclusion. To overcome this possibility more precise clinical evidence should be established by prospective clinical studies looking for variations of clinical manifestations.

In Russell's viper bite, the reported incidence of dry bite has been low, with most bites resulting in significant envenoming [8]. At the same time there were situations where, in proven cases of Russell's viper bite, acute renal failure was the sole manifestation of envenoming developing many hours after the bite [18]. From the clinicians' perspective, early recognition of significant systemic envenoming is of great importance as it would enable taking an early decision regarding antivenom therapy. At present there is no early sign or symptom that has been identified in Russell's viper bite that could be considered a clinical predictor of significant systemic envenoming. Abdominal pain has been documented as a clinical feature in some studies of Russell's viper envenoming [19], [20]. But abdominal pain is also a known clinical feature of envenoming by other snakes, particularly by the common krait [21]. However, epidemiology of common krait bite is unique that it happens exclusively at night when victims are in sleep causing neuromuscular paralysis. Nevertheless, many practicing clinicians believe that abdominal pain is a feature of systemic envenoming in Russell's viper bite (personal communication, Dr. W P Dissanayake, consultant physician). This observation is strengthened by the fact that in a comparative study of the patients of Russell's viper and Hump-nosed pit viper (*Hypnale* spp.) bites, abdominal pain was present only among the Russell's viper bite patients [20].

These issues indicated that there was justification for revisiting the clinico-epidemiology of Russell's viper bites in Sri Lanka, using a well designed prospective clinical study in order to broaden the present understanding of the clinical profile and analysing the place of abdominal pain as an early feature of significant envenoming. The objectives of the present study therefore are to re-evaluate the clinico-epidemiology of Russell's viper envenoming in Sri Lanka and to examine the place of abdominal pain as a possible early clinical feature of significant envenoming.

## Materials and Methods

### Ethical background

Ethical clearance for this study was obtained from the Ethics Review Committee of the Faculty of Medicine, University of Peradeniya, Sri Lanka. Written informed consent was obtained from the participants in this study prior to data collection. Proxy consent was obtained from the relatives of those who were unconscious and of those below the age of 18 years.

### Study setting

The study was carried out in the Emergency Treatment Unit (ETU) of the Teaching Hospital, Anuradhapura (THA), from January to December 2010.

THA is a tertiary care centre and the largest hospital in the North Central Province and indeed, in the entire dry zone of Sri Lanka, from where the highest number of snakebites is reported annually [4]. The hospital receives patients from a large catchment that consists mostly of rural paddy (rice) and *chena* (slash and burn) farming areas.

### Data collection

Patients who were admitted to the hospital with a history of snakebite were screened and those with confirmed Russell's viper bites authenticated with the positive identification of the offending snake and suspected Russell's viper bite patients were enrolled in the study. Data collection was prospective using investigator administered questionnaires. Information regarding patients with altered consciousness and about children was obtained from relatives, parents or those in attendance. IBM SPSS version 20.0 was used for the data analysis.

### Snake identification

The Department of Wildlife Conservation, Sri Lanka, permitted (by permit letter no: WL/3/2/1/7) the retention of the killed snakes brought along with snakebite victims that were then taken to the University of Peradeniya where the identification was done using identification keys in De Silva, 1980. In addition, whenever the assailant snake specimen is not available for identification, preserved specimens and photographs of Russell's vipers were shown to the patients for the tentative establishment of the identity of the assailant snake. However these cases were considered as suspected cases of Russell's viper bite.

### Clinical assessment

The severity of envenoming was assessed using the scoring system described in table 1 where each manifestation, namely local envenoming, coagulopathy, neuromuscular paralysis, renal dysfunction, cardiovascular dysfunction and abdominal pain was given a score on a cumulative scale. Each manifestation could score a minimum of 1 point with additional points depending on increasing severity of involvement. Severity of antivenom reactions were assessed retrospectively as mild, moderate or severe based on clinical parameters: normal blood pressure with cutaneous manifestation was categorized as a mild reaction; dropping of systolic blood pressure below 90 mm Hg was considered a moderate reaction; and patients who had cardiovascular collapse with unrecordable blood pressure and thready pulse were considered to have a severe reaction.

**Table 1:** The scoring system adopted to grade the severity of envenoming and the severity of abdominal pain.

Description of the clinical manifestation category	Severity score
**Abdominal pain**	
Occurs within 6 hours of bite	1
Onset within 30 minutes of bite	0.5
Lasting more than 1 hour	0.5
Bearable with difficulty/Unbearable	0.5
**Local envenoming**	
Presence of local swelling	1
Swelling involving more than half the limb	1
Necrosis/Gangrene/Compartment syndrome	1
**Coagulopathy**	
On admission 20WBCT prolonged (>20 min)	1
Subsequent 20WBCTs prolonged (repeated 6 hourly, if any)	1
Spontaneous bleeding (local bleeding/gum bleeding/epistaxis/subconjunctival haemorrhage	1
Severe bleeding (gasterointestinal bleeding/haematuria)	1
**Neuromuscular paralysis**	
Ptosis/Opthalmoplegia	1
Neck/limb muscle weakness	1
Respiratory paralysis	1
**Renal dysfunction**	
Blood urea >13 mmol/l	1
Oliguria with high blood urea nitrogen/high serum K^+^	1
Renal failure requiring renal replacement therapy	1
**Cardiovascular dysfunction**	
Systolic blood pressure <90 mmHg and signs of early circulatory collapse	1
Severe circulatory collapse (shock)/arrhythmias	1
Acute myocardial infarction/heart failure	1

## Results

### Demographic and epidemiological data

**Figure 2:**
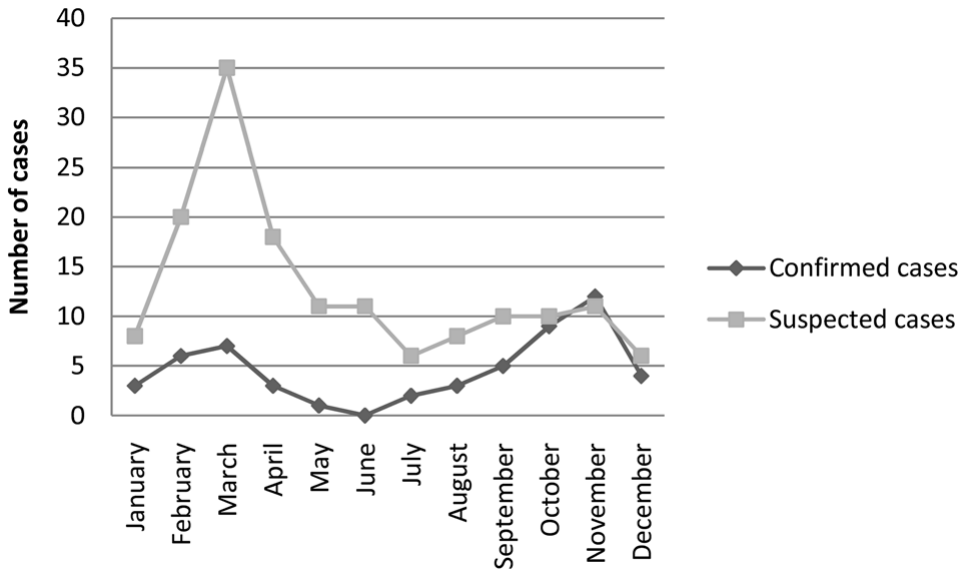
Seasonal variation of Russell's viper bites. Distribution of the confirmed and suspected cases of Russell's viper bites admitted to the Teaching Hospital, Anuradhapura from January to December, 2010. Note the high rate of admissions during February – April and September – November representing paddy harvesting and chena cultivation seasons.

Of the study group, 92.7% patients directly came to the THA. During the study period, 55 specimen-authenticated (proven) and 154 suspected Russell's viper bite patients were treated in the study setting and were thus included in the study. The majority of victims were in the 20–50 year age group: 72.7% and 76.6% in the proven and suspected Russell's viper bite groups respectively. Most affected were males (81% and 84%) and farmers (45% and 67%) in both groups. However, a range of other occupations, including security personnel, school teachers, businessmen and school children were seen among the participants of this study. The vast majority (88% and 93%) of the Russell's viper bite patients in the two groups had received school education only up to grade 11 or age 16. Figure 2 shows the seasonal distribution of these patients. There are high rates of Russell's viper bites during February to May and September to December, the paddy (rice) harvesting and chena cultivating seasons in the dry-zone of Sri Lanka. Bites had occurred in chenas (53% and 66%), paddy fields (5.5% and 13%), in and around residential compounds (20% and 11%) and by the roadside (20% and 9%) among the proven and suspected Russell's viper bite patients. In the above two groups, 67% and 61% of all bites had occurred during the period 12.00–21.00 hours, respectively.

### First aid treatment

Washing the bite site (62%, 51%) and application of a tourniquet proximal to the bite site (42%, 57%) were the commonest first aid measures practiced prior to hospital admission in proven bites and suspected Russell's viper bites respectively. No first aid had been administered to 22% of the proven Russell's viper bite victims and 0.65% of the others. The median time interval between the snakebite and hospital admission was 28 and 30 minutes (range 10–180 minutes) in proven and suspected Russell's viper bite victims respectively.

### Clinical features

The clinical findings of the study participants are described in table 2. Fifty-three (96%) of the proven Russell's viper bite patients had local or systemic envenoming, giving a dry bite rate of 4%, with 38 (69%) having systemic envenoming. All those with systemic envenoming also had local envenoming. Coagulopathy, characterized by prolonged clotting time (as determined by the twenty minute whole blood clotting test or 20WBCT) and spontaneous bleeding, was the commonest clinical manifestation of systemic envenoming among both groups. Neuro-muscular paralysis, renal toxicity and cardiovascular dysfunction were also seen in the study participants at varying degrees of severity and in various proportions (see table 2). These clinical manifestations occurred in isolation or in various combinations as described in table 3. The Spearman's rho test showed that the severity of each clinical manifestation did not correlate with the length of the snake (median  = 461 mm, range: 235–1320 mm) (table 4).

**Table 2:** Clinical findings of the 55 proven and 154 suspected Russell's viper bite patients. (percentages within parenthesis).

Clinical findings	Proven cases (n, 55)	Probable cases (n,154)
**Local envenoming at the site of bite**		
Total	49 (89)	143 (93)
Swelling	49 (89)	143 (93)
Pain	53 (96)	150 (97)
Blistering	1 (2)	0
Necrosis	1 (2)	0
**Coagulopathy**		
Total	38 (69)	121 (79)
Incoagulable blood (20 WBCT)	38 (69)	121 (79)
Spontaneous bleeding		
Gum bleeding	2 (4)	1 (0.65)
Epistaxis	0	2 (1)
Gastrointestinal bleeding	1 (2)	5 (3)
Haematuria	1 (2)	23 (15)
Time duration from bite to coagulopathy (Minutes, range within parenthesis)	167.5 (35–960)	165 (55–1050)
**Renal dysfunction**		
Total	5 (9)	34 (22)
Mild	3 (5)	18 (12)
Moderate	2 (4)	11 (7)
Severe (requiring renal replacement therapy)	0	5 (3)
**Neuromuscular paralysis**		
Total	30 (55)	116 (75)
Ptosis	30 (55)	109 (71)
Ophthalmoplegia	30 (55)	111 (72)
Neck muscle weakness	14 (25)	50 (32)
Respiratory paralysis	0	2 (1)
**Cardiovascular dysfunction**		
Total	1 (2)	11 (7)
Mild		10 (6.5)
Moderate		1 (0.65)

**Table 3:** Systemic involvement of the proven Russell's viper bite patients (n = 55) (percentages within parenthesis).

Description of the systemic involvement	Number of patients
Coagulopathy alone	7 (13)
Coagulopathy and Neuromuscular paralysis	27 (49)
Coagulopathy and Renal dysfunction	2 (4)
Coagulopathy, Neuromuscular paralysis and Renal dysfunction	2 (4)
Neuromuscular paralysis and Renal dysfunction	1 (2)

**Table 4:** Correlations between length of the snake versus different clinical parameters of 48 from the 55 confirmed Russell's viper bite patients.

Description	Correlation Coefficient	P value
Length of the snake	1.000	-
Envenoming severity	−0.084	0.572
Coagulopathy	−0.016	0.916
Neuromuscular paralysis	−0.006	0.966
Local envenomation	−0.189	0.199
Renal dysfunction	.005	0.971

(Statistical test -Spearman's rho.).

### Antivenom therapy

Antivenom therapy was administered to 64% and 75% of study participants with proven and suspected Russell's viper bites respectively. The first dose of antivenom had been administered at the THA in 94% and 90% respectively. The median time interval from bite to first dose of antivenom was 332 (range: 105–1090) and 287 (range: 105–1225) minutes in the proven and suspected groups respectively. The ranges of total antivenom vials and antivenom cycles required for proven and suspected Russell's viper bite are described in table 5. The median time taken for the whole blood clotting time to become normal was 778 minutes (range: 125–2070) and 589 minutes (range: 105–1200) among proven and suspected patient groups respectively. A mild reaction at least was observed in all those who received antivenom in both groups. Moderate or severe reactions were observed among 31% and 6% of proven and among 14% and 5% of suspected Russell's viper bite patients respectively.

**Table 5:** The numbers of antivenom vials and antivenom cycles received by the proven and probable Russell's viper bite patients.

	Proven cases (n, 55)	Probable cases (n, 154)
**Number of vials**		
10	2 (6)	4 (3)
15	0	2 (2)
20	22 (63)	64 (55)
25	0	1(1)
30	9 (26)	17 (15)
40	2 (6)	24 (21)
50	0	3 (3)
60	0	1 (1)
		
**Number of antivenom cycles**		
1	22 (63)	65 (56)
2	13 (37)	41 (35)
3	0	8 (7)
4	0	2(2)

(Percentages within parenthesis.).

### Abdominal pain

Abdominal pain was observed in 31 (79.5%) of the proven Russell's viper bite victims with systemic envenoming and in only 3 (25%) of those who had only local envenoming. The pain had developed 5 to 240 minutes (mean 71 minutes) after the bite and lasted up to the 2^nd^ (40.7%) or 3^rd^ day (18.2%), post bite. It was generalized in 52% of the patients, or localized to the umbilical (21%), epigastric (13%), supra-pubic (9%), right inguinal fossa (2.6%) or right lumbar (1.3%) region in others. The pain was described as colicky (60%), aching (32%), or burning (8%) in nature.

Abdominal pain co-existed with coagulopathy, neuromuscular paralysis and renal dysfunction in 78.9%, 80% and 60% of patients respectively. The Spearman's rank correlation coefficients between the abdominal pain scores and the respective severity scores of coagulopathy and neuromuscular paralysis (table 6) were highly significant. However, there were no statistically significant correlations between the abdominal pain scores and renal dysfunction or local envenoming. The analysis in terms of diagnostic validity of abdominal pain (table 7) found Sensitivity –81.6%, Specificity –82.4%, Positive predictive value –91.2%, False positives 3 (17.6%) and False negatives 7 (18.4%).

**Table 6:** Correlations between abdominal pain severity versus different clinical parameters of the 55 proven Russell's viper bite patients.

Severity score	Number of	Correlation Coefficient	P value
**Abdominal pain**			
1	14	1.000	-
1.5	11		
2	5		
2.5	1		
**Local envenoming**			
1	47	0.167	0.224
2	2		
3	0		
**Coagulopathy**			
1	24	0.461	<0.001
2	12		
3	2		
4	0		
**Neuromuscular paralysis**			
1	16	0.553	<0.001
2	13		
3	0		
**Renal dysfunction**			
1	3	0.018	0.894
2	2		
3	0		

(Statistical test -Spearman's rho.).

**Table 7:** Validation of abdominal pain against systemic envenoming*.

	No. of Patients with systemic envenoming	No. of Patients without systemic envenoming	Total
No. of patients with abdominal pain	31	3	34
No. of patients without abdominal pain	7	14	21
Total	38	17	55

* [Sensitivity –81.6%, Specificity –82.4%, Positive predictive value –91.2%, Negative predictive value –66.7%, False positives 3 (17.6%), False negatives 7 (18.4%)].

## Discussion

This study shows that Russell's viper bites are common among young and middle aged male farmers in Sri Lanka. The bite rates are high during the chena cultivation and paddy harvesting seasons, with the vast majority of these bites occurring in agricultural lands. These factors together make Russell's viper bites a definite occupational health hazard of farmers in Sri Lanka. However, the large number of patients representing a range of occupations other than farming highlights the burden of Russell's viper bites on Sri Lanka. Involvement of the lower limbs in nearly 90% of the patients emphasizes the preventability of these bites by wearing protective footwear during agricultural work and when walking on foot paths. Very high antivenom reaction rates observed in these patients highlights the vexed question of the safety of Indian polyvalent antivenom for use in Sri Lankan Russell's viper bite patients and the desperate, urgent need of safe and effective antivenom for use in Sri Lanka.

This study demonstrates that Russell's viper envenoming in Sri Lanka affects multiple systems in various combinations as well as mono-systemic involvement, leading to a variety of clinical syndromes. It also shows that abdominal pain is a common clinical feature of Russell's viper envenoming and its severity has a significant correlation to the severity of systemic envenoming with coagulopathy and neurotoxicity.

Syndromes of Sri Lankan Russell's viper envenoming consist of local swelling, incoagulable blood and spontaneous bleeding, acute renal failure and neurotoxicity in various combinations [8], [19]. Based on this observation, Ariaratnam et al. [17] developed an algorithm for identification of Russell's viper envenoming when the offending snake was not available. The signs included in the algorithm were combinations of neurotoxicity, incoagulable blood and renal failure. However, in our study, only 2 (4%) of the proven Russell's viper bite patients had all three systems involvement, raising a concern about the validity of the algorithmic approach as described above. Systemic involvement in Russell's viper envenoming seen in our study in rank order of frequency was as follows: coagulopathy and neurotoxicity; coagulopathy alone; coagulopathy and nephropathy; coagulopathy, neurotoxicity and nephropathy; neurotoxicity and nephropathy. Based on the results of our study, we emphasize the necessity of anticipating mono-systemic and possible combinations of multiple systemic effects in Russell's viper bite patients and it should be taken into consideration when making a clinical decision using syndromic approach.

The patient catchment of THA includes very remote areas, despite which 92.7% of all participants of this study were direct admissions. The median time interval from the incident to initiation of medical care was around 30 minutes, all the study participants having arrived within 3 hours, indicating that snakebite patients generally arrive quickly at a medical facility in Sri Lanka. The participants of this study received antivenom generally around 5 hours after the bite. The delay in commencing antivenom treatment following arrival in hospital, generally 4 ½ hours, was because of the time taken to confirm systemic envenoming by awaiting the appearance of specific clinical signs or the demonstration of coagulopathy by the 20WBCT.

Recently, the reliability of the 20WBCT performed in Sri Lankan hospitals for detecting Russell's viper bite induced coagulopathy has been questioned and reliance on clinical diagnosis of envenoming or the use of more accurate clotting studies over the 20WBCT suggested [22]. Prompt administration of antivenom following a bite resulting in systemic envenoming has the best chance of neutralising free venom and preventing the development of life-threatening situations. Delay in administration of antivenom, for example because the overt manifestation of clinical signs of systemic envenoming is delayed, reduces the chances of rapid venom elimination. In this situation the identification of early clinical features of severe Russell's viper envenoming would enable the early identification of the need for antivenom therapy. Abdominal pain, at present considered a non-specific symptom, is a likely candidate. It has long been neglected as a useful clinical sign of envenoming in Russell's viper bite patients in Sri Lanka. In developing the scoring system for syndromic identification of Russell's viper bite envenoming, abdominal pain has been taken as an unlikely clinical feature [16]. However, the results of the present study suggest the need of recognizing abdominal pain as an indicator of systemic envenoming due to Russell's viper bites, and an important observation on which to base the clinical decision regarding antivenom administration.

Russell's vipers and Hump-nosed pit vipers are responsible for most number of venomous snakebites in Sri Lanka [5]. Hump-nosed pit vipers commonly cause only local envenoming, less commonly, coagulopathy and renal failure [23] sometimes leading to a clinical picture somewhat similar to that of Russell's viper bites [20]. In about three-fourths of all snakebite instances in Sri Lanka, the offending snakes are not available for identification [7], [23]. The Indian polyvalent antivenom is not indicated for hump-nosed pit viper bites in Sri Lanka, therefore the ability to distinguish one clinically from an actual Russell's viper bite in such instances becomes important. The availability of the snake specimen also does not guarantee the accurate distinction between the two vipers in routine hospital practice in Sri Lanka [17]. In the circumstances the results of this study suggests that abdominal pain in a snake bite victim where the identity of the biting snake is unknown should weigh heavily in favour of antivenom administration or prompt transfer to a hospital where that can be done. This will minimise unnecessary delay in antivenom therapy when that is indicated as well as prevent the unnecessary administration of antivenom when it is not. However, it is prudent to take abdominal pain together with other clinical manifestations such as local pathology, non specific symptoms and early features of specific systemic manifestation to avoid risk of using a single parameter to judge the onset and severity of envenoming.

## Conclusion

Sri Lankan Russell's viper bites manifest as different combinations of system involvement as well as mono-system involvement, commonly isolated coagulopathy. It is felt that existing paradigms of syndromic approaches towards identifying Russell's viper envenoming needs careful re-assessment. Abdominal pain is a common, yet neglected early clinical feature in Russell's viper envenoming. Presence of abdominal pain and its severity is well correlated with the severity of coagulopathy, neurotoxicity and the severity of overall systemic envenoming, suggesting its use as a potential early clinical feature in Russell's viper bite envenoming in Sri Lanka.
